# Clinical Decision Support Systems and Artificial Intelligence in ADHD Assessment and Rehabilitation: Opportunities and Challenges for Technology-Assisted Care

**DOI:** 10.3390/healthcare13233171

**Published:** 2025-12-04

**Authors:** Margherita Dahò, Barbara Caci

**Affiliations:** 1 Department of Psychology, Educational Science and Human Movement, University of Palermo, Viale Delle Scienze, Ed.15, 90146 Palermo, Italy; barbara.caci@unipa.it; 2 WeSearch Lab–Laboratory of Behavioral Observation and Research on Human Development, University of Palermo, 90146 Palermo, Italy

**Keywords:** ADHD, artificial intelligence, clinical decision support systems, cognitive psychology, cognitive rehabilitation, decision-making, digital health, healthcare, human–computer interaction, personalized care

## Abstract

**Highlights:**

**What are the main findings?**

- AI-augmented Digital Clinical Decision Support Systems (CDSSs) have the potential to improve ADHD diagnosis, intervention planning, monitoring, and outcome prediction.
- Human–Computer Interaction (HCI) principles are essential for effective system design and user engagement in ADHD rehabilitation technologies.

**What are the implications of the main findings?**

- Integrating CDSSs and AI could enhance personalized ADHD care and optimize long-term functional outcomes.
- Ethical, practical, and implementation challenges must be addressed for successful adoption of technology-assisted ADHD interventions.

**Abstract:**

**Background**: Attention-Deficit/Hyperactivity Disorder (ADHD) is a prevalent neurodevelopmental condition affecting children, adolescents, and adults worldwide. Despite evidence-based treatments, long-term functional outcomes remain variable due to heterogeneity in symptoms, comorbidities, and environmental contexts. Digital technologies, including AI-augmented digital Clinical Decision Support Systems (CDSSs), are increasingly proposed to support more precise and personalized ADHD care. This concept paper provides a theoretical discussion of the potential applications of CDSSs in ADHD rehabilitation and examines key considerations for system design, usability, and ethical implementation. **Discussion**: CDSSs and AI technologies offer conceptual promise for enhancing ADHD care by integrating patient-specific data to guide diagnosis, intervention planning, monitoring, and outcome prediction. Incorporating Human–Computer Interaction (HCI) principles is critical to ensure systems are intuitive, engaging, and supportive of adherence, particularly for children and adolescents with ADHD. Ethical, practical, and implementation challenges, including data privacy, equity, and variability in healthcare infrastructures, must be addressed. Thoughtful design and governance of AI-supported CDSSs may improve decision-making, optimize functional outcomes, and facilitate more individualized rehabilitation pathways. **Conclusions**: The paper concludes by emphasizing future research directions that may include translating conceptual frameworks into empirically testable models, developing guidelines for user-centered and ethically responsible technology deployment, and evaluating long-term impacts on clinical outcomes. By providing a theoretical foundation, this paper aims to guide the integration of AI-augmented CDSSs into technology-assisted ADHD rehabilitation while highlighting the importance of ethical, practical, and human-centered design considerations.

## 1. Introduction

Attention-Deficit/Hyperactivity Disorder (ADHD) is a multifaceted neurodevelopmental condition marked by persistent patterns of inattention, hyperactivity, and impulsivity that emerge early in life and interfere with everyday functioning across settings [1]. Once regarded primarily as a childhood disorder, ADHD is now recognized as a condition that can persist across the lifespan, producing significant difficulties in academic achievement, interpersonal relationships, work performance, and overall well-being [1,2,3,4]. Global prevalence estimates range from 5% to 7% among children and from 2% to 6% among adults, reflecting both developmental continuity and diagnostic variability across the lifespan [5,6,7].

A defining characteristic of ADHD is disruption in executive functioning, which refers to the higher-order cognitive processes that enable planning, inhibition, cognitive flexibility, and the regulation of emotion and attention [4,8,9]. Deficits in these domains contribute to difficulties in sustaining goal-directed behavior, adapting to changing demands, and managing complex tasks [10]. Cognitive dysfunction, indeed, is not static: its expression evolves with development and varies considerably among individuals [11]. Therefore, while adolescents may struggle with self-organization and impulse control, adults usually encounter obstacles in managing responsibilities or maintaining motivation over the long term [12,13]. In later life, lingering executive challenges may also contribute to cognitive inefficiency and social withdrawal [3,14]. For these reasons, strengthening cognitive skills remains a central focus of ADHD intervention and rehabilitation throughout the lifespan [10]. Cognitive rehabilitation programs, particularly those targeting executive functions via structured exercises, compensatory strategies, and metacognitive training, have demonstrated efficacy [10,15,16]. However, translating theoretical models, such as those of Barkley [17] and Miyake and Friedman [18,19], into individualized interventions remains a challenge. Time constraints, limited resources, and a shortage of trained professionals, especially in low-resource settings, further restrict access [16]. Consequently, patients often face delayed assessments, uneven service distribution, and sometimes biased informal care [20,21]. As observed by Ishikawa [22], the increasing demand for ADHD services necessitates systemic strategies to expand clinical capacity and improve decision-making efficiency.

In this setting, clinicians must navigate a complex network of decisions, striking a balance between short-term symptom management and long-term functional goals. They must select among several behavioral, pharmacological, and cognitive interventions; determine intensity, duration, and sequencing; and adapt strategies to the patient’s motivation, age, and social environment. Overlapping symptoms with other disorders, evolving diagnostic frameworks, and variability in informant reports further complicate these decisions. These factors often lead to outcomes that rely heavily on clinical intuition, which limit the reproducibility, consistency, and scalability across settings [13,14,15]. Moreover, disagreements among members of the multidisciplinary care team can add to another layer of complexity, requiring negotiation and consensus-building.

Digital technologies, particularly Clinical Decision Support Systems (CDSSs), whether enhanced with AI or not, offer a promising strategy to address these challenges. CDSSs are designed to integrate heterogeneous clinical data, identify patterns, and provide evidence-informed guidance tailored to individual patients [23,24]. By synthesizing information from multiple sources, such as cognitive assessments, behavioral observations, and treatment history, these systems can support clinicians in making more consistent, transparent, and personalized decisions [25,26]. Furthermore, CDSSs can facilitate ongoing monitoring, dynamically adjusting interventions based on patient response and engagement, and can assist multidisciplinary teams in aligning on intervention strategies. In this way, technology-assisted platforms have the potential to enhance both the efficiency and the quality of ADHD rehabilitation while reducing reliance on subjective intuition [27]. Notably, the successful adoption of CDSSs requires careful attention to Human–Computer Interaction (HCI) principles to ensure that tools are usable, engaging, and aligned with the cognitive and emotional needs of both patients and clinicians [28]. HCI principles guide the design of intuitive interfaces, minimize cognitive load, provide clear and actionable feedback, and adapt presentation styles to individual users [28]. By complementing rather than replacing human expertise, CDSSs can also translate complex clinical data into actionable guidance, support decision-making under uncertainty, and provide continuous, personalized feedback on interventions [23,24,26]. Through this integration of computational models and clinician expertise, such tools can thus promote more consistent, transparent, and scalable rehabilitation strategies [27].

### Aims of the Study

This concept paper provides a comprehensive overview of ADHD as a complex clinical and rehabilitative challenge, highlighting the potential of CDSSs and AI to enable more adaptive, personalized, and patient-centered care. It examines the methodological, practical, and ethical considerations associated with implementing these technologies and situates them within the broader context of cognitive rehabilitation. To enrich this discussion, the paper also reviews and compares major national and international clinical guidelines for ADHD management, identifying key areas of convergence and divergence in treatment approaches across healthcare systems. In this way, it aims to inform future research, guide the development of safe and evidence-based interventions, and promote human-centered solutions that support clinicians, patients, and multidisciplinary teams in managing ADHD effectively.

## 2. Decision Support Systems and AI in Healthcare and Cognitive Rehabilitation

As briefly introduced, CDSSs are computational tools designed to assist clinical decision-making by integrating data, generating recommendations, and predicting outcomes [24]. In healthcare, these systems have been used to support diagnostic processes, risk assessment, and treatment planning, e.g., [23,26,29,30,31,32,33,34,35,36,37,38]. By integrating clinical guidelines with real-time data, CDSSs provide clinicians with structured guidance, facilitate evidence-based decision-making, and consistent care delivery, e.g., [23,24,35]. For instance, Diraco et al. [38] developed a system to guide multisensory stimulation therapy in dementia, demonstrating how CDSSs can help clinicians tailor interventions by selecting appropriate sensory modalities and adjusting therapeutic intensity according to individual patient profiles. Similarly, digital CDSS platforms have been employed in cognitive rehabilitation to personalize exercises and track user performance over time, e.g., [27,39,40]. These systems dynamically adjust task difficulty, select appropriate cognitive modules, and provide real-time feedback based on patient progress and adherence, thereby promoting continuity of care, reducing clinician workload, and enabling more objective tracking of therapeutic outcomes [27]. Finally, systematic reviews report that these systems improve nursing performance, guideline adherence, and patient outcomes, particularly in areas like anticoagulation management, glycemic control, and triage [26,32,37].

CDSSs can also vary considerably in their technological sophistication: some rely primarily on rule-based algorithms and expert-validated guidelines, whereas others integrate AI components. The contrast between non-AI and AI-driven approaches highlights the spectrum of CDSS development, from structured, guideline-based systems to adaptive, learning tools that evolve with clinical data. For instance, the Beacon Decision Support System (Beacon DSS [30,41]) is a non-AI platform designed to promote equity in oncology by providing clinicians, patients, researchers, and policymakers with centralized, validated, and accessible information resources. The Beacon DSS, developed within the BEACON project (Bridging European Access to Cancer Oncology Networks), was funded under the EU4Health program (Grant Agreement Number: 101080005) and implemented by the technical partner Sporedata Inc (Tallinn, Estonia). From a technical standpoint, Beacon DSS integrates heterogeneous controlled data streams (such as clinical guidelines, trial databases, outcome registries, reimbursement frameworks, etc.) into a unified access layer, employs standardized ontologies for semantic harmonization, and exposes API-based services to allow secure retrieval, query, and embedding of oncology knowledge into external clinical or research workflows. Unlike AI-based recommender systems, Beacon DSS does not generate predictions or automated treatment suggestions; instead, it operates as a curated evidence infrastructure intended to support transparency, comparability, and equitable access to validated oncology information across national contexts. Its usability evaluations highlighted both its potential to improve transparency and its challenges in personalization and search efficiency, underscoring the importance of user-centered design and stakeholder engagement in CDSS development [42]. Similarly, NOVA-L (Navigating Options & Vital Assistance for Life-limiting conditions [31]) has been proposed as a conceptual, family-centered CDSS designed to empower caregivers, facilitate informed decision-making, and complement clinical care in neonatal and pediatric settings. It is technically conceived with principles such as the Beacon DSS, which provides access to validated information rather than AI-generated recommendations, and an emphasis on transparency, equity, and decision support rather than automation. Such principles are equally relevant to ADHD management, where variability in guidelines, care accessibility, and population needs similarly calls for transparent, adaptable, and evidence-based digital tools.

In contrast, AI-enhanced CDSSs employ machine learning to analyze large datasets, detect subtle patterns, and generate adaptive, data-informed recommendations. Specifically, deep learning, a subfield of machine learning, utilizes artificial neural networks composed of multiple hierarchical layers to represent, interpret, and manage complex data structures [43]. Moreover, multimodal data acquisition can yield extensive heterogeneous datasets encompassing demographic characteristics, cognitive performance scores, neuropathological markers, vital parameters, clinical manifestations, medical histories, neuropsychological test batteries, and laboratory findings e.g., [44,45]. Such comprehensive datasets can be exploited to develop advanced predictive frameworks, including CDSSs aimed at promoting early diagnostic assessment and continuous disease monitoring within primary or routine clinical care environments [44]. Among the various deep learning architectures that can be integrated into CDSSs, Convolutional Neural Networks (CNNs) have demonstrated exceptional efficacy in analyzing neuroimaging data [46]. CNNs are exceptionally proficient in identifying spatial hierarchies and structural relationships within images, rendering them highly effective for processing brain imaging modalities such as magnetic resonance imaging and positron emission tomography [46]. When embedded in decision support systems, CNNs can autonomously learn discriminative patterns linked to neuropathological conditions, such as Alzheimer’s disease, thereby supporting clinicians in early detection, improving diagnostic precision, and enabling longitudinal monitoring of cerebral changes [45].

Additional examples of AI-driven CDSSs in healthcare include ePAL [35], which is designed to optimize pain management for cancer patients in palliative care settings. The system collects patient-reported outcomes via structured questionnaires and longitudinal symptom tracking; it uses predictive models to generate individualized treatment suggestions and alert clinicians when symptom patterns deviate from expected trajectories. In this way, it offers personalized recommendations based on patient-reported outcomes gathered through structured questionnaires and ongoing symptom tracking. Similarly, PreCare (Advanced Care Planning Assistant [34]) is an AI-supported, web-based application that guides patients and their families through advanced care planning, facilitating reflection on personal values, informed decision-making, and knowledge acquisition. In addition to structured, value-elicitation modules, the system uses natural-language-based recommender logic to tailor educational content and decision prompts to the user’s stage of understanding and expressed preferences, while preserving clinician oversight.

To conclude, these systems demonstrate that integrating AI with human-centered design, through continuous patient input, adaptive content delivery, and model-driven, clinician-supervised recommendations, can enhance reliability, reinforce clinical judgment, and foster trust in AI-augmented decision-making. More broadly, these examples illustrate the transformative potential of CDSSs to bridge the gap between research and practice by translating evidence into actionable, patient-centered guidance. Despite these advances, however, CDSSs specifically designed for ADHD rehabilitation remain limited. This gap thus underscores the need for systems capable of addressing disorder-specific challenges, including variability in symptom presentation, developmental differences, and the influence of contextual factors such as learning environments and family dynamics [47]. Applying these principles to ADHD rehabilitation could thus promote more dynamic, individualized, and equitable approaches to treatment planning and cognitive support.

## 3. ADHD Treatment Frameworks: EU vs. US Guidelines and Unmet Challenges

By presenting CDSSs in detail first, we highlighted the importance of embedding accurate, evidence-based guidance into these systems. This motivates the present section, which reviews ADHD treatment frameworks in the United States and across some European countries. Summarizing these guidelines serves two purposes. First, it provides the empirical and procedural foundation necessary for developing a CDSS that integrates validated, internationally recognized recommendations. Second, it underscores the degree of variability that clinicians must navigate when making treatment decisions across different healthcare contexts. Indeed, ADHD management is governed by multiple overlapping frameworks that differ not only between the U.S. and Europe but also among European member states. These variations concern diagnostic thresholds, preferred treatment modalities, the sequencing of behavioral and pharmacological interventions, and access to specialist care. For example, while U.S. guidelines typically favor medication as the first-line treatment for school-age children with moderate to severe symptoms, many European guidelines adopt a more cautious, stepwise approach that prioritizes behavioral or psychosocial interventions, particularly for younger children [47,48]. Beyond philosophical differences in treatment orientation, disparities also arise from the organization of the healthcare system, reimbursement models, and the availability of trained professionals [47]. Such diversity thus presents a significant challenge for both clinical practice and digital innovation. For clinicians, it means that decision-making is rarely straightforward: local policies, resource constraints, and patient preferences must all be reconciled with evidence-based standards. For technology developers, it means that CDSSs must be designed with flexibility to accommodate multiple guideline sources, adapt to contextual differences, and remain transparent about their recommendation logic.

To capture these differences, the following subsections summarize general ADHD treatment recommendations from the United States and selected European countries, including France, Germany, Italy, Portugal, Spain, and the U.K., highlighting areas of convergence, divergence, and ongoing debate. Finally, a comparative table (Table 1) provides an overview of these national frameworks.

### 3.1. Guidelines Overview in the United States

In the U.S., the American Academy of Pediatrics [49] and the American Psychiatric Association [50] recommend pharmacological treatment, often stimulant medications, as a first-line intervention for school-age children with moderate to severe ADHD symptoms [49,50]. The AAP guideline, in particular, emphasizes that medication dosing should be carefully titrated to balance efficacy and side effects, and urges clinicians to monitor treatment response and adverse events over time [49]. However, even in the U.S. setting, key challenges persist around adherence and individualization. Studies indicate that many patients do not maintain consistent use of medication over time. For example, Charach et al. [51] describe multiple barriers to medication adherence in ADHD, including side effects, perceived lack of efficacy, forgetfulness, cost issues, and stigma. More recent work suggests that only a portion of children and adolescents adhere to prescribed regimens long enough to reap durable benefits [52]. Additionally, treatment must be tailored to individual needs because ADHD is heterogeneous in presentation, comorbidities, and developmental stage. However, psychosocial interventions are also presented as essential adjuncts, especially parent training, behavioral classroom management, and school-based interventions [48,49]. The guideline itself acknowledges that behavioral strategies and pharmacotherapy should often be combined and that interventions must be individualized based on severity, comorbidities, family preferences, and response [49]. To provide practitioners and developers with a reliable source, the complete AAP guideline is publicly available on the AAP website, and it includes detailed recommendations for age groups (preschool, school-age, and adolescents), titration schedules, monitoring protocols, and the incorporation of behavioral support.

### 3.2. European Guidelines and Country-Level Variability

Across Europe, clinical guidelines for ADHD broadly emphasize a multimodal and developmentally sensitive approach, with behavioral and psychosocial interventions generally recommended as first-line treatments for children and adolescents, and pharmacotherapy reserved for more severe or persistent cases [48,53]. However, practical implementation varies widely across countries, reflecting differences in healthcare organization, access to specialized services, and cultural attitudes toward medication. Countries with well-structured national frameworks, such as Germany and Italy, promote comprehensive strategies that integrate psychoeducation, behavioral therapy, and school-based support alongside pharmacological interventions, when indicated [54]. In Germany, adherence to prescribed treatment varies by region, reflecting the impact of local infrastructure, clinician training, and family engagement on outcomes. Similarly, in Italy, ADHD management is primarily guided by multiple key documents from the Italian Society of Childhood and Adolescent Neuropsychiatry (SINPIA), including the 2002 clinical guidelines on diagnosis, psychotherapy, and pharmacological treatment [55,56] and the National Consensus Conference guideline [57] on therapeutic strategies. These documents advocate family-centered, multimodal care and structured assessment processes. Nevertheless, regional disparities persist, particularly for adult ADHD services, which remain unevenly distributed and can limit consistent access to specialized care [58].

Other European contexts, including France, Spain, Portugal, and the U.K., illustrate further variability in approach [59,60]. Indeed, although multidisciplinary and individualized approaches are recommended, variations in service availability and healthcare resources frequently determine treatment patterns and adherence. For example, the UK NICE guideline [53] no longer recommends parent training as first-line treatment for school-aged children due to lower effect sizes and quality of evidence compared to pharmacotherapy, although parent training remains first-line for preschool-aged children (<5 years); while in Spain, approved drugs may be used without a specific recommended priority [61]. Across these contexts, long-term adherence remains a challenge, reflecting broader difficulties in maintaining consistent treatment over time, even in countries with well-structured national frameworks [62,63].

This cross-national variability also highlights that ADHD management is influenced not only by structural, cultural, and logistical factors but also by the populations most frequently targeted by existing guidelines. European frameworks largely converge on the value of multimodal care, yet much of the literature and clinical guidance remains focused on children and adolescents [64]. Guidance for adults, and especially older adults, is comparatively sparse, reflecting gaps in evidence, service provision, and specialized training across member states [64,65].

As mentioned above, we have summarized all this information in Table 1 to visually represent the comparison among countries more effectively.

**Table 1:** Comparison of ADHD Treatment Guidelines in Select Countries.

Country	National Guidelines	Pharmacotherapy First-Line	Behavioral/Psychoeducational Support
France	[59]	Considered after a multidisciplinary assessment.	Neuropsychological and educational support
Germany	[54]	Typically, after behavioral interventions, stimulant medications are considered first-line.	Structured parent training is recommended
Italy	[55,56,57]	Usually, after behavioral interventions, pharmacotherapy is considered second-line.	Parent training, psychoeducation, psychotherapy (variable)
Portugal	[60]	Typically, behavioral interventions are first; pharmacotherapy is considered if necessary.	Parent training and school-based support
Spain	[61]	Often, behavioral interventions are first; pharmacotherapy is considered if necessary.	Psychoeducation, parent training
UK	[53]	If the child is >5 and symptoms are severe, stimulant medications are first-line	Parent training, behavioral support
USA	[49,50]	Typically, pharmacotherapy and stimulant medications are first-line.	Behavioral interventions adjunct to pharmacotherapy

## 4. Potential Applications of DSS for ADHD

As highlighted in previous paragraphs, managing ADHD involves navigating complex, multifactorial decisions influenced by developmental stage, cognitive profile, comorbidities, and contextual factors such as healthcare system structure and cultural norms. Digital technologies, particularly CDSSs enhanced with AI, offer a promising avenue to address these challenges by integrating heterogeneous data, synthesizing clinical guidelines, and generating personalized recommendations [24,66].

While the available literature on the current state of DSS applications for ADHD is limited and insufficient for a complete narrative or systematic review, the few studies identified provide a valuable foundation for defining potential directions for research, design, and clinical integration. These studies were identified through a brief literature search conducted in October 2025 using the terms “ADHD” AND (“DSS” OR “Decision Support System” OR “CDSS” OR “Clinical Decision Support System”) across PubMed, Google Scholar, and PsycNet; after removing duplicates and including only empirical studies with specific ADHD populations, four studies were included. Building on the insights of these studies, we organized the discussion into three subsections corresponding to the primary areas of CDSS application in ADHD. These subsections examine how CDSSs can: (A) support diagnostic and screening processes by synthesizing clinical data and identifying risk or symptom patterns; (B) aid intervention planning and monitoring, integrating pharmacological, behavioral, and cognitive rehabilitation strategies in line with evidence-based guidelines; and (C) enable outcome prediction and the creation of individualized care pathways, tailoring recommendations to developmental stage, cognitive profile, and contextual factors. (For sub-section (C), no empirical studies specific to ADHD are currently available; instead, we discuss potential applications and speculate on future uses based on evidence from broader child and adolescent mental health literature.)

### 4.1. Diagnostic and Screening Support

CDSSs can support the diagnostic process and early screening of ADHD by integrating multiple sources of information, including teacher and parent questionnaires, performance on computerized cognitive tasks, and data from wearable sensors [67]. These systems enable risk stratification and identify potential “red flags” for timely referral, facilitating the more efficient identification of individuals who require comprehensive assessment, particularly in resource-limited settings. Moreover, CDSSs designed for adult ADHD diagnosis have demonstrated their utility in improving workflow, standardizing evaluation procedures, and enhancing consistency in clinical decision-making [68]. For instance, Kemppinen et al. [68] implemented a CDSS integrated into the regional electronic health record, named Effica, in a Finnish hospital to address the frequent underdiagnosis of adult ADHD. The system was co-designed with a multiprofessional team (psychiatrists, psychologists, ADHD nurses, occupational therapists, and social workers) to enforce a common, structured diagnostic pathway. Beyond decision support, the system, although not AI-based, enabled real-time tracking of process bottlenecks, resource constraints, and time consumption across the care pathway, and facilitated rapid process adjustments. Reported outcomes included gains in diagnostic efficiency and a marked increase in productivity (e.g., from approximately 2.6 to 4.6 patient-related visits per day after process optimization). The CDSSs thus supported clinicians by structuring patient information, standardizing reporting, and identifying bottlenecks in the diagnostic process, ultimately improving both efficiency and accuracy.

Similarly, other studies have explored the use of computerized decision aids for ADHD diagnosis in pediatric populations. For instance, Carroll et al. [67] conducted a randomized controlled trial involving 84 medical charts from two intervention and two control clinics. The study evaluated the CHICA (Child Health Improvement through Computer Automation) system, an electronic health-record–integrated CDSS. CHICA automatically triggers Vanderbilt questionnaires (i.e., standardized rating scales completed by parents and teachers to assess ADHD symptoms [69]) when initial screening flags are positive. In addition, the system generates clinician prompts aligned with evidence-based guidelines and produces tailored parent handouts. The study found that the use of digital structured diagnostic assessments for ADHD increased from 60% to 81% in the intervention group, compared to a decrease from 50% to 38% in the control group, even after controlling for age, gender, and race. This difference was statistically significant, indicating that the computerized decision aid improved adherence to diagnostic guidelines and increased the consistency of clinician assessments.

Finally, Chu et al. [70] developed the DS-ADHD system, a decision-support system designed to improve the reliability and validity of ADHD diagnoses. The study included 107 participants, divided into ADHD and non-ADHD groups based on DSM-IV clinical assessment. Participants completed both the Test of Variables of Attention (TOVA [71]) and the DS-ADHD assessment. Statistical analyses, including one-way ANOVA and split-half reliability tests, showed that DS-ADHD had higher diagnostic accuracy than TOVA, with a receiver operating characteristic area of 0.867 (95% CI: 0.801–0.933) compared to 0.787 (95% CI: 0.701–0.872) for TOVA. The system thus integrates multiple sources of patient data and guides clinicians through structured assessment pathways, supporting more objective, transparent, and consistent diagnostic decisions. However, while DS-ADHD does not explicitly use AI or machine learning, its design exemplifies intelligent decision support by analyzing complex data to assist clinicians in making informed diagnoses.

Taken together, these first findings illustrate that CDSSs can serve as a valuable adjunct to clinical judgment, particularly in contexts where ADHD symptoms are complex, presentations are heterogeneous, and standardized assessment is critical. By consolidating information from multiple sources and applying evidence-informed algorithms, such systems may support clinicians in making more objective, transparent, and consistent diagnostic decisions.

### 4.2. Intervention Planning and Monitoring of Cognitive Rehabilitation Programs

CDSSs can also play a pivotal role in optimizing ADHD interventions over time by providing dynamic, data-driven guidance to clinicians and patients. These systems can support clinicians in adjusting medication regimens based on observed responses and reported side effects, enabling personalized, iterative treatment planning. Beyond pharmacological management, CDSSs can enhance adherence to behavioral and cognitive rehabilitation programs by delivering digital prompts, reminders, or gamified feedback, thus fostering engagement and consistency in therapy.

An illustrative example is the empirical study by Bashiri et al. [27], which developed an adaptive CDSS for cognitive rehabilitation in children with ADHD. Participants included 95 children and adolescents with ADHD, aged 7–18 years, referred to the Parand and Aren centers from 2013 to 2016. The system integrates data from cognitive assessments, such as attention and response control scores from the Integrated Visual and Auditory test [72] to select and tailor computerized training programs for individual users. A total of 28 parameters were extracted from the IVA-CPT in both visual and auditory dimensions for each participant, and scores below 90 were considered indicative of deficits. The selected programs included Captain’s Log, a structured cognitive training platform designed to improve attention, memory, and executive function [73]; Lumosity, a widely used brain-training suite with games targeting processing speed, working memory, and problem-solving [74]; and Maghzineh, a program focused on enhancing attention, planning, and cognitive flexibility [75]. Through expert input and if–then rule modeling in MATLAB [76], the platform dynamically adjusted task difficulty, type, and timing based on the child’s performance and engagement. The system allowed therapists to enter individual IVA-CPT parameters and receive a set of recommended exercises for each detected deficit, with program names linked directly to the application or website, and visual outputs showing the recommended exercises for specific cognitive domains such as selective attention.

In sum, while CDSSs for ADHD remain relatively limited, this work illustrates how adaptive, rule-based digital systems can complement clinical judgment. By combining patient-specific data, evidence-based exercises, and real-time performance feedback, these platforms can provide tailored, developmentally appropriate interventions that address disorder-specific challenges, such as variability in symptom presentation, executive dysfunction, and fluctuations in engagement [27].

### 4.3. Outcome Prediction and Individualized Care Pathways

The predictive capabilities of CDSSs are currently hypothetical in the context of ADHD and can inform individualized care planning by estimating likely responses to different interventions, anticipating comorbid risks, and projecting long-term functional outcomes [25]. By combining predictive modeling with prescriptive recommendations, we suggest that CDSSs can guide decisions about when to intensify, adjust, or step down interventions, constructing adaptive care pathways tailored to each patient’s cognitive profile and life context. While CDSSs specifically developed for outcome prediction in ADHD are lacking, evidence from a broader systematic review in pediatric psychiatry by Koposov et al. [25], which also included ADHD, indicates their potential usefulness. For instance, the myGRaCE DSS [77] was developed to support collaborative assessment and management of mental health risks, particularly related to suicide, self-harm, harm to others, self-neglect, and vulnerability. The system was designed through an iterative process involving 115 service users and clinicians, combining interviews, focus groups, and agile software development to elicit user requirements and refine usability. By integrating service user and practitioner expertise, myGRaCE produces structured yet flexible assessments that dynamically adjust to user input. Its stepwise evaluation process incorporates continuous feedback at each stage, offering a foundation for predictive insights into emerging risks and for guiding timely intervention strategies. Furthermore, integration with the GRiST web-based platform (the Galatean Risk and Safety Tool, a clinical decision support system for mental health services [77,78]) allows alignment between self-assessments and professional evaluations, enhancing data-driven, personalized mental health management.

Similarly, the Net Decision Support System (NetDSS [33]) was developed to facilitate evidence-based collaborative care for depression and to support care managers in tracking and predicting progress within primary care settings. The system implements clinical protocols derived from randomized controlled trials through a web-based interface, providing structured patient registries, encounter scheduling, and automated outcome reporting that enable longitudinal monitoring of patient trajectories. Through embedded decision rules and progress indicators, the NetDSS provides feedback loops that help clinicians anticipate suboptimal responses and refine intervention intensity [33]. Having been used to manage depression care for over 1700 primary care patients, the system demonstrated how predictive and prescriptive functionalities can sustain high-fidelity, adaptive care pathways in real-world contexts.

Together, these examples underscore how CDSSs can move beyond static decision support to anticipatory, data-informed guidance, laying conceptual groundwork for future predictive systems tailored to ADHD rehabilitation and treatment monitoring.

## 5. Ethical, Practical, and Implementation Challenges

While CDSSs hold promise for improving ADHD care, their integration into clinical practice raises a series of intertwined ethical, practical, and technical considerations. At the heart of these concerns lies the tension between leveraging large-scale data for personalized recommendations and ensuring that these recommendations are trustworthy, equitable, and clinically meaningful. Data quality and representativeness are indeed critical, as many ADHD datasets underrepresent variations in age, comorbidities, and cultural context, which can inadvertently perpetuate disparities if system outputs are applied uncritically [29,79]. Similarly, AI-driven algorithms, particularly opaque “black-box” models, can challenge both clinician and patient trust if the reasoning behind recommendations is not transparent [66,79].

Beyond these issues, integrating AI into clinical decision-making raises additional ethical and professional implications. One primary concern is the potential for overreliance and clinical deskilling: as AI tools become increasingly capable of producing detailed, plausible recommendations, clinicians, especially those early in their careers or working under pressure, may be tempted to accept automated suggestions without sufficient critical reflection [24,80,81]. Over time, such dependence risks weakening essential clinical autonomy and competencies such as individualized formulation, flexible treatment planning, and therapeutic reasoning. Moreover, excessive reliance on automated output risks diminishing clinician autonomy and critical engagement [24,80,81].

Equally relevant is the issue of contextual and cultural insensitivity. Since many AI systems are trained on large but homogeneous datasets, they may fail to account for differences in sociocultural background, language, family context, or environmental factors. Consequently, without careful human oversight, these tools may unintentionally reinforce existing inequities or overlook the needs of underrepresented populations [82,83,84]. Moreover, AI systems lack the ethical sensitivity and emotional intelligence required for many clinical judgments. Decision-making involves informed consent, patient autonomy, or family involvement and demands empathy, moral reasoning, and contextual understanding—all dimensions that remain uniquely human [82,85,86].

Successful implementation also depends on institutional safeguards: secure infrastructure, interoperability with electronic health records, clinician training, and precise governance mechanisms that delineate professional accountability. Establishing transparent boundaries between AI-assisted decision-making and autonomous clinical judgment is vital to prevent ethical ambiguity or diffusion of responsibility, particularly in cases of adverse outcomes [87,88]. Furthermore, CDSSs must guarantee data privacy, transparency, and explainability, ensuring that both clinicians and patients, especially when minors are involved, can understand and trust the system’s outputs [89,90]. AI-driven CDSSs, in particular, face challenges related to data bias, algorithmic opacity, and the risk of generating misleading recommendations [91].

Despite these challenges, AI offers substantial potential in ADHD care. As seen, it can analyze large datasets, identify subtle behavioral patterns, and provide personalized feedback, which may be especially valuable given the heterogeneity and comorbidities that often complicate treatment decisions. At the same time, these possibilities highlight the need for a human-centered and ethically informed approach to the development and implementation of CDSSs. Furthermore, ethical reflection cannot be separated from clinical and practical realities: only by ensuring transparency, adaptability, and sensitivity to diverse developmental and contextual factors can these systems truly improve the quality and equity of care [89]. Finally, it is important to note that poor decisions can occur at any time, whether made by clinicians, by AI, or by a combination of both [92]. This reality thus underscores the need to strike the right balance between human judgment and AI support, establish careful oversight, and clearly define responsibilities to safeguard patient trust, reduce disparities, and prevent harm [21].

## 6. Conclusions and Future Directions

This concept paper provided a descriptive overview of the potential of clinical decision support systems in ADHD rehabilitation, emphasizing approaches that are adaptive, personalized, and informed by HCI principles. While the technological capabilities of CDSSs in ADHD are promising, their ultimate effectiveness depends on careful design, empirical validation, and ethical implementation.

Future research should prioritize co-design and pilot prototyping with end users, including both clinicians and patients, to ensure usability, engagement, and relevance to real-world practice. In addition, longitudinal studies are needed to evaluate sustained outcomes, adherence, and usability across diverse populations. Comparative trials assessing CDSS-augmented interventions against standard care can also help identify best practices and clarify where AI support is most beneficial. Importantly, the current literature remains limited in several areas. There is a notable gap in research on CDSS applications for adults and older adults with ADHD, as well as systems that systematically address comorbidities ranging from depression and anxiety to dementia. Investigating these populations could provide critical insights into how CDSSs can accommodate complex clinical profiles, enhance personalized care, and mitigate health disparities. Furthermore, research should explore the potential of hybrid models that integrate clinician expertise with AI-driven insights, striking a balance that maximizes decision accuracy while preserving professional judgment. Broader studies could also examine how lessons learned in ADHD rehabilitation may generalize to other neurodevelopmental and psychiatric conditions, supporting the development of cross-condition, scalable CDSS solutions. Finally, establishing clear guidelines for transparency, accountability, and integration within clinical workflows will be essential to ensure safe, ethical, and effective deployment.

To conclude, ADHD offers a valuable context for advancing the development of CDSSs, serving not only as a model for technology-assisted rehabilitation but also as a platform for innovations that could be applied to other clinical populations, especially those with complex or comorbid conditions.

## Data Availability

No new data were created or analyzed in this study. This study is based entirely on previously published literature.

## Abbreviations

The following abbreviations are used in this manuscript:

AI	Artificial Intelligence
ADHD	Attention-Deficit/Hyperactivity Disorder
AAP	American Academy of Pediatrics
APA	American Psychiatric Association
CDSSs	Clinical Decision Support Systems
CHICA	Child Health Improvement through Computer Automation
DGPPN	Deutsche Gesellschaft für Psychiatrie und Psychotherapie, Psychosomatik und Nervenheilkunde
GRIST	Galatean Risk and Safety Tool
GPC-TDAH	Grupo de trabajo de la Guía de Práctica Clínica sobre las Intervenciones Terapéuticas en el Trastorno por Déficit de Atención con Hiperactividad
HAS	Haute Autorité de Santé
HCI	Human Computer Interaction
NICE	National Institute for Health and Care Excellence
SINPIA	Italian Society of Childhood and Adolescent Neuropsychiatry
